# Lebensbedrohliche Einsatzlagen: Handlungsempfehlung zur Zusammenarbeit zwischen Polizei, Kliniken und nichtpolizeilicher Gefahrenabwehr

**DOI:** 10.1007/s00101-024-01434-0

**Published:** 2024-07-25

**Authors:** E. G. Pfenninger, T. O. Hammer, T. Holsträter, S. Weiß

**Affiliations:** 1 Stabsstelle Katastrophenschutz, Universitätsklinikum Ulm, Ulm, Deutschland Albert-Einstein-Allee 29, 89081; 2 Stabsstelle Katastrophenschutz, Risiko- und Gefahrenabwehr Universitätsklinikum, Freiburg, Deutschland; 3 Abteilung für Anästhesiologie, Intensivmedizin, Notfallmedizin und Schmerztherapie, Bundeswehrkrankenhaus Ulm, Ulm, Deutschland; 4 Zentralbereich Katastrophenschutz RKH Kliniken Ludwigsburg, Ludwigsburg, Deutschland

**Keywords:** Terrorismus, Kommunikation, Führungs- und Lagezentrum, Präklinisches Management, Klinische Versorgung, Terrorism, Communication, Command and control center, Prehospital management, Clinical care

## Abstract

**Hintergrund:**

Die Gefahr terroristischer Anschläge in der Bundesrepublik Deutschland ist gegeben und nimmt aktuell weiter zu. Öffentlich geförderte Akutkrankenhäuser haben eigenverantwortlich umfassende Vorsorge für ihre Einsatzfähigkeit bei Katastrophen zu treffen. Dieser Auftrag ist auch bei Terror- und Amoklagen sicherzustellen. Eine optimale Abstimmung zwischen präklinischer und klinischer Versorgung ist unabdingbar.

**Fragestellung:**

Es werden Handlungsempfehlungen zur Zusammenarbeit von außerklinischer und klinischer Planung zur Bewältigung eines Massenanfalls von Verletzten bei lebensbedrohlichen Einsatzlagen (LebEL) vorgestellt.

**Material und Methoden:**

Die Klinikübergreifende Sicherheitskonferenz Baden-Württemberg (KLÜSIKO BW) ist eine Arbeitsgemeinschaft aus Vertretern der Akutkliniken in Baden-Württemberg, des Ministeriums für Inneres, Digitalisierung und Migration Baden-Württemberg, des Ministeriums Soziales und Integration Baden-Württemberg, des Landespolizeipräsidiums Baden-Württemberg und der Baden-Württembergischen Krankenhausgesellschaft e. V. Von 2018 bis 2020 wurden in der KLÜSIKO BW Handlungsempfehlungen zur „Zusammenarbeit zwischen Polizei, Kliniken und nichtpolizeilicher Gefahrenabwehr“ erarbeitet. Die Handlungsempfehlungen wurden in 6 Arbeitssitzungen konzertiert und in 2 anschließenden Videokonferenzen parafiert. Eine Empfehlung galt als verabschiedet, wenn abschließend die Vollversammlung der KLÜSIKO BW ihre Zustimmung mit absoluter Mehrheit gab.

**Ergebnisse und Diskussion:**

Zu fordern sind kompetenzbasierte Schnittstellenlösungen für ein reibungsloses Zusammenwirken von Präklinik und Klinik bei der Versorgung von Patienten, die Opfer einer LebEL wurden. Für die Vorplanung wird die Einrichtung einer lokalen Sicherheitskonferenz auf der Ebene Landkreis/Untere Katastrophenschutzbehörde mit folgenden Teilnehmern empfohlen: Untere Katastrophenschutzbehörde, Feuerwehr, regionales Polizeipräsidium, Leitender Notarzt, Rettungsdienste, Katastrophenschutzbeauftragte betroffener Kliniken. Empfohlen wird ein gemeinsames Führungs- und Lagezentrum (FLZ), wo sich Führungspersonal von Polizei, Rettungsdienst, Feuerwehr und Katastrophenschutz einfinden, um die Abwicklung des Schadensereignisses gemeinsam, kompetent und ohne Zeitverlust zu organisieren. Aus diesem FLZ sollten dann die Kliniken in regelmäßigen zeitlichen Abständen mit Informationen versorgt werden. Übungen sollten regelmäßig stattfinden. Eine besondere Bedeutung kommt organisationsübergreifenden Übungen zu.

## Abstract (en)

**Background:**

The risk of terrorist attacks in the Federal Republic of Germany is present and is currently increasing. Publicly funded acute care hospitals and their owners are involved in disaster control as part of their remit and are responsible for taking comprehensive precautions to ensure their operational capability in the event of disasters. This mandate must also be ensured in the event of terrorist attacks and amok incidents. For this purpose, an optimal cooperation between preclinical and clinical care is indispensable.

**Aim:**

Recommended actions for collaboration between nonclinical and clinical planning to manage a mass casualty incident in terrorist life-threatening response situations are presented.

**Material and methods:**

The Inter-Hospital Security Conference Baden-Württemberg (IHSC BW) is an association of representatives of acute hospitals in Baden-Württemberg, the Ministry of the Interior, Digitalization and Migration Baden-Württemberg, the Ministry of Social Affairs and Integration Baden-Württemberg, the State Police Headquarters Baden-Württemberg and the Baden-Württemberg Hospital Association. From 2018 to 2020, the IHSC BW developed recommendations for action on cooperation between police, hospitals and non-police emergency response. The recommendations for action were agreed by the group members in 6 working sessions and initialled in two subsequent video conferences. A recommendation was considered adopted when the IHSC BW plenary assembly finally gave its approval with an absolute majority.

**Results and discussion:**

Competence-based interface solutions for a smooth cooperation between prehospital and hospital management in the care of patients who have become victims of a terrorist attack are to be demanded. For preliminary planning, the establishment of a local safety conference at the county disaster control authority level with the following participants is recommended: disaster control authority, fire department, regional police headquarters, chief emergency physician, rescue services and disaster control officers of affected clinics. It is recommended to set up a joint command and situation center (CSC), where management personnel from the police, rescue service, fire department and disaster control can meet to organize the handling of the incident jointly, competently and without loss of time. From this CSC, a liaison officer should then provide the clinics with information at regular intervals. Exercises should take place regularly. Cross-organizational exercises are particularly important, and this is one of the tasks of the local safety conference.

## Einleitung

Die Gefahr terroristischer Anschläge in der Bundesrepublik Deutschland ist gegeben und kann noch weiter zunehmen [1]. Die Bundesregierung hat kürzlich ein Dokument zur nationalen Sicherheitsstrategie vorgelegt, in dem die Bedeutung des Katastrophenschutzes sowie der Abwehr terroristischer Bedrohungen betont wird [2], zudem warnte ganz akut der Verfassungsschutz Ende 2023: „die Gefahr von Terroranschlägen … sei so hoch wie lange nicht mehr“ [3]. Hier ist auch die veränderte geopolitische Lage zu berücksichtigen, indem eine neue Bedrohungslage bis hin zu einer asymmetrischen Kriegsführung droht. Krankenhäuser zählen zu den kritischen Infrastrukturen, die per definitionem als Organisationen und Einrichtungen mit wichtiger Bedeutung für das staatliche Gemeinwesen anzusehen sind [4]. Öffentlich geförderte Akutkrankenhäuser und ihre Träger wirken gemäß § 5 Abs. 1 des Landeskatastrophenschutzgesetzes (LKatSG) im Rahmen ihres Aufgabenbereichs am Katastrophenschutz mit und haben eigenverantwortlich umfassende Vorsorge für ihre Einsatzfähigkeit bei Katastrophen zu treffen [5].

Gemäß § 28 Absatz 2 des Landeskrankenhausgesetzes Baden-Württemberg (LKHG) stellen die Krankenhäuser „[…] durch geeignete Vorkehrungen, insbesondere durch die Erstellung und Fortschreibung von Alarm- und Einsatzplänen sicher, dass auch bei einem Massenanfall von Verletzten oder Erkrankten eine ordnungsgemäße Versorgung der Patienten gewährleistet werden kann“ [6]. Dieser Auftrag ist auch bei Terror- und Amoklagen sicherzustellen [7]. Terror- und Amoklagen werden nach aktuell gültiger Definition unter dem Begriff „lebensbedrohliche Einsatzlage (LebEL)“ subsumiert.

Ein Massenanfall an Verletzten (MANV) liegt dann vor, wenn die aktuell zur Verfügung stehenden Versorgungs- und Behandlungskapazitäten für das durch eine Gefahren- oder Schadenslage verursachte Aufkommen von Verletzten überschritten werden und damit ein Ressourcenmangel droht oder eingetreten ist. Szenarien bei Anschlägen haben gezeigt, dass sich diese weitgehend von der Situation eines „normalen“ MANV unterscheiden, es wurde deshalb der Begriff Massenanfall von Verletzten in lebensbedrohlichen Einsatzlagen (MANV bei LebEL) für diese Art von Ereignissen eingeführt [5–8]. In vielen Publikationen wird der Begriff TerrorMANV synonym zu der Begrifflichkeit MANV bei LebEL verwendet. Die Unterschiede vom MANV bei LebEL zum „normalen“ MANV bestehen zum einen in der Tatsache, dass es sich um völlig andere Verletzungsmuster, wie Schuss- und Explosionsverletzungen, handelt [9]. Dominierend sind hierbei perforierende und penetrierende Verletzungen, die regelhaft eine schwere Hämorrhagie verursachen, weshalb eine rasche und unmittelbare Blutungskontrolle angestrebt werden muss [8]. Zum anderen sind diese Patienten aus logistischen Gründen in der Regel in der ersten Phase eines MANV bei LebEL präklinisch unversorgt, sodass aus Sicht der Klinik eine neue zusätzliche Herausforderung darin besteht, eine Erstversorgung der Patienten innerklinisch durchführen zu müssen [9]. Diese organisatorische Aufgabe wird vorwiegend von Anästhesisten und Unfallchirurgen wahrgenommen. Erschwerend kommt hinzu, dass bei der Bewältigung eines LebEL verschiedene Behörden und Organisationen mit Sicherheitsaufgaben (BOS) im Sinne der koordinierten Versorgung zusammenarbeiten müssen, die unterschiedlichen Aufbau und Führungsstrukturen aufweisen [10]. Bezeichnend ist hierzu, dass am 19.04.2023 die Europäische Kommission die zweite Stufe im Vertragsverletzungsverfahren u. a. gegen Deutschland wegen unzureichender Umsetzung der Richtlinie (EU) 2017/541 zur Terrorismusbekämpfung beschlossen hat [11].

Sowohl in der nationalen wie auch in der internationalen Literatur sind weder evidenzbasierten Daten noch Guidelines zur außerklinisch-innerklinischen Zusammenarbeit bei LebEL zu finden. Die im Folgenden dargelegten Handlungsempfehlungen sind konzertierte Eckpunkte zur Zusammenarbeit von außerklinischer und klinischer Planung der Bewältigung eines Massenanfalls von Verletzten bei LebEL, die von der Klinikübergreifenden Sicherheitskonferenz Baden-Württemberg (KLÜSIKO BW) erarbeitet wurden.

## Material und Methode

Die KLÜSIKO BW wurde im Juni 2018 in Ulm gegründet. Sie ist ein Zusammenschluss aus Vertretern der Akutkliniken in Baden-Württemberg, des Ministeriums für Inneres, Digitalisierung und Migration Baden-Württemberg, des Ministeriums Soziales und Integration Baden-Württemberg, des Landespolizeipräsidiums Baden-Württemberg und der Baden-Württembergischen Krankenhausgesellschaft e. V. [12]. Laut Gründungssatzung sind die Ziele der KLÜSIKO BW Informations- und Erfahrungsaustausch bei der Vorbereitung von Akutkrankenhäusern auf den Massenanfall von Verletzten bei Terrorlagen sowie Erarbeitung von Standards zu Sicherheitsaspekten für die Kliniken in Terror- und Amoklagen.

In 4 Arbeitsgruppen wurden von 2018 bis 2020 Handlungsempfehlung zur „Zusammenarbeit zwischen Polizei, Kliniken und nichtpolizeilicher Gefahrenabwehr“ erarbeitet. Die Handlungsempfehlungen wurden in 6 Arbeitssitzungen von den Gruppenmitgliedern konzertiert und in 2 anschließenden Videokonferenzen parafiert. Eine Empfehlung galt als verabschiedet, wenn abschließend die Vollversammlung der KLÜSIKO BW ihre Zustimmung mit absoluter Mehrheit gab.

## Ergebnisse und Diskussion

1. *Zielsetzung und Definition*
  *Die* „*Zusammenarbeit zwischen Polizei, Kliniken und nichtpolizeilicher Gefahrenabwehr“ stellt Rahmenempfehlungen und Handlungshinweise dar, die die Zusammenarbeit der vorgenannten Organisationen bei lebensbedrohlichen Einsatzlagen (LebEL) regeln.*
  *LebEL sind zunächst nicht eindeutig klassifizierbare Einsatzlagen mit hohem Gefährdungspotenzial für das Leben von Opfern, Unbeteiligten und Einsatzkräften, bei denen ein oder mehrere Täter insbesondere mittels Waffen, Sprengmitteln, gefährlichen Werkzeugen bzw. Stoffen oder außergewöhnlicher Gewaltanwendung gegen Personen vorgehen, diese verletzt oder getötet haben und weiter auf Personen einwirken können. Eine LebEL im polizeitaktischen Sinn liegt bereits vor, wenn Anhaltspunkte ein solches Täterverhalten unmittelbar erwarten lassen *[13]*.*
  *Gemäß den Hinweisen des Ministeriums für Inneres, Digitalisierung und Migration für die nichtpolizeiliche Gefahrenabwehr bei Einsätzen im Zusammenhang mit Terror- oder Amoklagen vom 02.08.2017 – Az.: 6‑1502.0/2 sind in den Einsatzplanungen Schnittstellen zu den Krankenhäusern zwingend zu berücksichtigen *[7]*.*

Organisation und Verletztenmanagement am Katastrophenort bei terroristischen Anschlägen werden in einer Vielzahl von Publikationen thematisiert [14–17]. Andere Veröffentlichungen beschreiben das Verletztenmanagement in den Kliniken [4, 9, 18, 19]. Öffentlich geförderte Akutkrankenhäuser und ihre Träger wirken im Rahmen ihres Aufgabenbereichs am Katastrophenschutz mit und haben eigenverantwortlich umfassende Vorsorge für ihre Einsatzfähigkeit bei Katastrophen zu treffen [6]. Dieser Auftrag ist auch bei Terror- und Amoklagen sicherzustellen [7]. In länderministeriellen Anweisungen ist zu finden, dass in den Einsatzplanungen Schnittstellen zu den Krankenhäusern zwingend zu berücksichtigen sind [7]. Rettungsdienst und Katastrophenschutz und damit deren Planungen sind in den meisten Bundesländern in den Innenministerien angesiedelt [7, 20], wohingegen die innerklinischen Zuständigkeiten bei Sozial- oder Gesundheitsministerien liegen [21]. Hieraus können sich bei der Planung zur Bewältigung terroristischer Anschläge Schnittstellenprobleme ergeben [8]. So gibt es kaum Publikationen zu gemeinsamen Übungen, die sowohl das Geschehen an der Unglückstelle als auch die nachfolgende Versorgung der Verletzten in Kliniken beschreiben [10]. Eine durchgeführte gemeinsame außer- und innerklinische Übung zur Versorgung von Terroropfern zeigte, dass an der Schnittstelle zwischen Präklinik und Klinik erhebliche kommunikative und organisatorische Mängel bestehen. So wurden bei dieser Übung 2 der 3 zur Aufnahme der Patienten vorgesehenen Kliniken erst nach 35 und 140 min nach dem simulierten Terroranschlag verständigt, die dritte Klinik, die die zweitmeisten Verletzten aufnehmen sollte, wurden dagegen überhaupt nicht informiert. Nur 65 % der vorgesehenen Patienten wurden in die Kliniken eingewiesen; von 16 Schwerstverletzten wären vermutlich 44 % wegen verzögerten Transportzeiten verstorben [10]. Schon bei der regulären notfallmedizinischen Versorgung ist die Schnittstelle zwischen Präklinik und Klinik ein kritischer Punkt, der der besonderen Beachtung bedarf [22]. Zeit- und Informationsverlust können zur vitalen Gefährdung des Patienten führen bzw eine vitale Gefährdung so weit verzögern, sodass ein Überleben des Verletzten nicht mehr gewährleistet ist. Klare Absprachen über Hierarchie, Zuständigkeiten und Behandlungspfade können das Gefährdungspotenzial in der Versorgung von Terroropfern minimieren [22]. Hierzu ist ein strukturiertes Alarm- und Anmeldesystem zwischen Präklinik und Klinik unerlässlich.

- *Um den Sicherstellungsauftrag gem. § 28 Abs. 2 LKHG auch bei LebEL sicherstellen zu können, ist es geboten, dass im Rahmen der Planung vorbereitender Maßnahmen ein Informationsaustausch zwischen den Kliniken, der Polizei und der nichtpolizeilichen Gefahrenabwehr erfolgt. Insbesondere die zu erwartenden Verletztenmuster sind frühzeitig zu kommunizieren. So ist in den genannten Einsatzlagen davon auszugehen, dass aufgrund einer großen Anzahl an amputations- beziehungsweise schussverletzten Personen ein hoher Anteil an Operationsindikationen zu erwarten ist *[7]*.*

Aufgaben und Ziele der Versorgung von Verletzten bei einer LebEL sind die Rettung möglichst vieler Opfer und deren unter den Umständen bestmögliche medizinische Versorgung. Die optimale Zusammenarbeit von Kliniken, Rettungsdienst sowie BOS ist dabei essenziell. Durch unterschiedliche Zuständigkeiten, Befugnisse, Führungsstrukturen und Organisation können jedoch immer wieder Reibungspunkte wahrgenommen werden [8]. „Aus diesem Grund sollte jede Führungskraft über Grundkenntnisse der benachbarten BOS, insbesondere hinsichtlich deren Aufgaben, Zuständigkeiten und Befugnisse verfügen, um Kompetenzstreitigkeiten vorzubeugen“ [8].

Beim Eintritt eines Massenanfalls von Verletzten (MANV), bei einem Terroranschlag mit vielen Verletzten oder anderen Großschadenslagen sollten Kliniken von den Rettungsdienstleitstellen oder der Integrierten Leitstelle (IL) aus Rettungsdienst und Feuerwehr rechtzeitig informiert werden [10, 22]. „Für die Information der Krankenhäuser stehen in einigen Bundesländern zusätzlich IT-gestützte Systeme (z. B. IVENA) oder spezielle Alarmfaxe zur Verfügung. Der Amoklauf in München und der Terroranschlag am Breitscheidplatz in Berlin haben zudem die hohe Bedeutung der sozialen Medien (z. B. Twitter) gezeigt. Über die sozialen Netzwerke erhalten Krankenhäuser oft schon vor der offiziellen Alarmierung Kenntnis und Informationen über ein mögliches Ereignis.“ [22]. Sinnvoll hingegen wäre es, ein gemeinsames Führungs- und Lagezentrum (FLZ) einzurichten, das z. B. bei der Polizei angesiedelt ist, und wo sich Führungspersonal von Polizei, Rettungsdienst, Feuerwehr und Katastrophenschutz einfinden, um gemeinsam, kompetent und ohne Zeitverlust die Abwicklung des Schadensereignisses zu organisieren [24]. In ministeriellen Hinweisen zur nichtpolizeilichen Gefahrenabwehr bei Einsätzen im Zusammenhang mit Terror- oder Amoklagen findet sich: „Der Rettungsdienst entsendet geeignete Verbindungspersonen in den Führungsstab der einsatzführenden Behörde …und in die Einsatzleitung vor Ort. Die Verbindungsperson besitzt die Qualifikation eines Leitenden Notarztes und wird durch einen Organisatorischen Leiter Rettungsdienst (OrgL-RD) unterstützt“ [7]. Aus diesem FLZ sollte dann ein „Verbindungsbeamter“ die Kliniken in regelmäßigen zeitlichen Abständen mit Informationen versorgen. Dies betrifft sowohl das Geschehen, die Anzahl Verletzter, die Anzahl und Zeit voraussichtlich in der Klinik ankommender Patienten sowie die Art und Schwere ihrer Verletzungen.

Gerade bei Terroranschlägen können sich Art und Schwere der Verletzungen erheblich vom Verletzungsmuster eines „normalen“ MANV, wie es z. B. bei Verkehrsunfällen zu finden ist, unterscheiden. So stellt nach Friemert et al. [9, 15] neben der unkalkulierbar hohen Anzahl an Verletzten der durch Explosionen oder andere penetrierende Verletzungen verursachte hämorrhagische Schock erhebliche logistische Anforderungen an die aufnehmende Klinik. Frühzeitig muss deshalb in einer Klinik die Entscheidung bei der Versorgung der Verletzten zugunsten von „life before limb“ getroffen werden, indem in den sog. „Tactical-abbreviated-surgical-care“-Modus geschaltet wird (TASC-Modus, [15]). Da es nicht sinnvoll ist, dass nur einzelne Bereiche einer Klinik in diesem Modus arbeiten, sondern alle Bereiche der Klinik, sind zur Entscheidungsfindung unbedingt suffiziente Informationen aus dem FLZ notwendig.

Nach Solarek [23] hat sich dabei für die Alarmierung folgendes Vorgehen in der Klinik bewährt:

- „Lageinformation: Erreichbarkeit sicherstellen; Katastrophenbeauftragten informieren; Krankenhausvorbereitung überprüfen
- Voralarmierung: Medizinischen Einsatzleiter (MEL) etablieren; Sichtung etablieren; Registrierung vorbereiten; OP-Programm überprüfen und ggf. anpassen; Personalsituation überprüfen
- Alarm: Krankenhauseinsatzleitung (KEL) einberufen; Notaufnahme, OP, Versorgungsbereiche, Intensivstationen sowie Servicebereiche verstärken; Entscheidung über externe Personalalarmierung treffen; ggf. Aufnahmekapazität rückmelden“ [23].
  - *Den Leitstellen wird empfohlen, stets aktuelle und zeitverlaufsabhängige Übersichten der Krankenhäuser über die Aufnahmekapazitäten in Abhängigkeit der zu erwartenden Verletzungsmuster einzuholen. Zur Unterstützung ist ereignisabhängig [soweit vorhanden] eine Oberleitstelle einzubinden. Auf deren Aufgaben – insbesondere in den Bereichen „Führen einer landesweiten Übersicht einschließlich der Erreichbarkeiten“ sowie „Führen einer zentralen Übersicht über die Akutkrankenhäuser“ – wird an dieser Stelle verwiesen. Ein dynamischer, elektronischer Bettennachweis, der mindestens landesweit verfügbar ist, sollte implementiert werden *[7].

Idealerweise ist die Aufnahmekapazität aller Krankenhäuser in die Einsatzleitrechner der Rettungsleitstellen integriert. Die Ad-hoc-Abfrage von Bettenkapazitäten bei einem MANV oder einem TerrorMANV entweder durch die Integrierte Leitstelle oder die Klinikeinsatzleitung eines Krankenhauses (KEL) im Ereignisfall ist allerdings wenig zielführend, da sie personal- und zeitintensiv sowie fehleranfällig ist [24]. Zudem können aus zeitlichen Gründen kaum die unterschiedlichen Versorgungsmöglichkeiten reflektiert werden. Kürzlich wurde in der Literatur ein sog. Wellenplan vorgestellt. Darin wurden in einem Umkreis von 100 km um Ulm potenziell aufnehmende Kliniken befragt, wie viele Patienten mit welchem Schweregrad an Verletzungen sie ad hoc aufnehmen könnten. Die Kliniken versicherten, dass sie die rückgemeldeten Kapazitäten zur Verfügung stellen, die Patienten können somit ohne weitere Rückfrage transferiert werden [25]. Baden-Württemberg unterhält eine Oberleitstelle, die „die Integrierten Leitstellen in Baden-Württemberg bei besonderen Einsätzen wie z. B. bei einem Massenanfall von Patienten [unterstützt]. Zur Vorbereitung dieser Aufgabe als Alarmzentrale erhält sie … Informationen über die strukturelle Behandlungs- und Bettenkapazität der Akutkrankenhäuser“ [26]. Allerdings ist nicht bekannt, in welcher Weise diese Daten erhoben werden, wie aktuell sie sind, und ob die tatsächlichen Aufnahmekapazitäten eruiert werden. Zudem werden nur Krankenhäuser des Landes Baden-Württemberg gelistet. Einige große Universitätsklinika z. B. haben ihr Einzugsgebiet nicht nur in Baden-Württemberg, sondern, da sie in relativer Nähe zu anderen Bundesländern oder gar zum Ausland liegen, werden im Bedarfsfall Patienten grenzüberschreitend transferiert werden.

Schon seit längerer Zeit wird die Einführung eines Krankenhauskatasters in den Klinikalltag für Großschadens- und Bedrohungslagen gefordert. Die Autoren schreiben: „Hierzu müssen alle Krankenhäuser ihre Aufnahmekapazitäten bei Großschadenslagen für Patienten nach den Sichtungskategorien SK I, SK II und SK III für ‚sofort‘ und nach ‚Hochfahren des Krankenhausalarms‘ in diesem Kataster hinterlegen … So könnte sinnvollerweise festgelegt werden, dass jedes Traumazentrum in der Akutphase in der Lage sein muss, mindestens bis zu 10 Patienten innerhalb der ersten Stunde aufzunehmen … Nach Hochfahren des Krankenhausalarms müsste dann die doppelte Menge an Patienten aufgenommen werden“ [27]. Die Forderung nach einer fixen Festlegung der Aufnahmekapazität ist theoretischer Natur und gewährleistet nicht, dass sie im Akutfall auch tatsächlich realisiert werden kann. Zudem scheinen die geforderten Zahlen willkürlich gegriffen und nicht an die örtlichen Versorgungsmöglichkeiten adaptiert zu sein. So sollte z. B. gelistet sein, welche Kliniken neurochirurgische oder pädiatrische Versorgungskapazitäten bereitstellen können [25]. Eine aktuelle und zeitverlaufsabhängige Übersicht der Krankenhäuser über die Aufnahmekapazitäten in Abhängigkeit der zu erwartenden Verletzungsmuster könnte die Situation deutlich verbessern.

Seit 2013 steht das webbasiertes IT-System Interdisziplinärer-Versorgungsnachweis (IVENA) zur koordinierten Verteilung von Patienten zur Verfügung. Das IVENA-System sammelt grundlegende Informationen (Alter und Geschlecht des Patienten, erwartete Diagnose (z. B. „Handtrauma“) und medizinisches Fachgebiet, Triage-Kategorie, medizinisches Transportsystem und voraussichtliche Ankunftszeit) und weist die Patienten je nach Nähe und Verfügbarkeit einem Krankenhaus zu [28]. Wenn ein Patient zugewiesen wird, erhält das Krankenhaus in Echtzeit eine webbasierte Benachrichtigung. Allerdings hängt die tatsächliche Aufnahmekapazität von der „Meldewilligkeit“ der beteiligten Kliniken ab. Eine regelmäßige flächendeckende Kapazitätenplanung der im Bedarfsfall kurzfristig zur Verfügung stehenden Betten würde hier eine deutlich Planungssicherheit bieten.

2. *Struktur und Organisation der Einsatzleitungen*
  *Eine gegenseitige Sensibilisierung der beteiligten Organisationen ist unabdingbar.*
  *Für die Vorplanung wird die Einrichtung einer lokalen Sicherheitskonferenz auf der Ebene Landkreis/Untere* *Katastrophenschutzbehörde empfohlen. Die Sicherheitskonferenz soll in regelmäßigen Abständen mit folgenden Teilnehmern stattfinden:*
    *Untere Katastrophenschutzbehörde (Leitung der Sicherheitskonferenz)*
    *Vertreter der Feuerwehr (Kreisbrandmeister, Leiter der Berufsfeuerwehr oder ggf. Feuerwehr-Kommandant)*
    *Vertreter des örtlich zuständigen regionalen Polizeipräsidiums*
    *Leitender Notarzt (LNA) (Sprecher d. LNA-Gruppe bzw. LNA im Bereichsausschuss)*
    *Vertreter des Rettungsdienstes*
    *Katastrophenschutzbeauftragter (oder Äquivalent) jeder Klinik im Zuständigkeitsbereich*
  *Da LebEL in der Regel überregionale Auswirkungen haben, wird empfohlen, in der Vorplanung eine landkreisübergreifende Planung speziell im Bereich des Rettungsdienstes und Katastrophenschutzes durchzuführen.*

Während im politischen Geschehen [29] oder auch in der Wirtschaft Sicherheitskonferenzen durchaus üblich sind, hat dieser Gedanke als übergreifende Institution im medizinischen Bereich noch wenig Einzug gehalten. Am Universitätsklinikum Ulm wurde 2017 eine „Klinikübergreifende Sicherheitskonferenz“ gegründet, in der die drei großen Kliniken, Polizei, Feuerwehr, Katastrophenschutz und Rettungsdienst in regelmäßigen Sitzungen das gemeinsame Vorgehen bei LebEL abstimmen. Als Vorteil ist anzusehen, dass die wesentlich Verantwortlichen der einzelnen Organisationen sich durch die gemeinsame Planung persönlich kennen und damit auch eine vertrauensvolle Zusammenarbeit im Ernstfall gewährleistet ist. Zusätzlich sollte die Einsatzplanung interdisziplinär in der Umsetzung beübt werden [7], denn gemeinsame Planungsbesprechungen und daraus abgeleitete Übung gewährleisten das Erkennen und Beheben von Planungsfehlern: „Einsatzplanung [soll] … auch im Rahmen einer interdisziplinären Regelkommunikation als Diskussionsgrundlage dienen, um aktuelle Informationen und Entwicklungen auszutauschen und ggf. Anpassungen in der Einsatzplanung vornehmen zu können“ [7].

In § 14 des Rettungsdienstgesetzes (RDG) des Landes Baden-Württemberg, „Grenzüberschreitender Rettungsdienst“ ist zu finden: „Das Innenministerium trifft mit anderen Bundesländern, mit Trägern des Rettungsdienstes oder sonstigen Stellen außerhalb von Baden-Württemberg Vereinbarungen, wenn dies zur Gewährleistung einer wirksamen Durchführung des Rettungsdienstes zweckmäßig ist“ [30]. Ähnliche Formulierungen finden sich auch in den Rettungsdienstgesetzen anderer Bundesländer. Leider zeigt sich aber immer wieder, dass die Zusammenarbeit bezüglich der Planung zwischen den einzelnen Bundesländern verbesserungswürdig ist. So sind z. B. Ulm und Neu-Ulm 2 nur durch die Donau getrennte Städte, jedoch liegen sie auch in 2 verschiedenen Bundesländern. Zwar arbeiten Kliniken und Rettungsdienst in der täglichen Praxis länderübergreifend gut zusammen, dies ist jedoch bezüglich Katastrophenplanung durchaus nicht so. Hier sollten, gerade wenn bei einer Katastrophe präklinisch und klinisch Patienten bundesländerübergreifend schnell und unbürokratisch versorgt werden müssen, gemeinsame Planungen angestrebt werden.

- *Seitens der jeweiligen Polizeipräsidien soll den in deren Zuständigkeitsbereich ansässigen Kliniken ein entscheidungsbefugter Ansprechpartner für die Besondere Aufbauorganisation (BAO) LebEL benannt werden.*
- *Die Einsatzleitungen aller beteiligten Organisationen sollten in einem Stabsmodell, wie in den Dienstvorschriften von Polizei und Feuerwehr (PDV 100, FwDV 100) beschrieben, aufgebaut sein.*

Die Kommunikation zwischen Polizei und medizinischen Einrichtungen spielt eine entscheidende Rolle bei der Bewältigung von Terroranschlägen [31]. Die Herausforderungen in der Kommunikation können durch eine effiziente Vorabplanung sowie klare Übermittlungsprotokolle bewältigt werden. Definierte Protokolle für die Übermittlung von Informationen können die kommunikative Effizienz erhöhen und die Wahrscheinlichkeit von Fehlern verringern. Kliniken benötigen rechtzeitige Informationen über Ausmaß des Terroranschlags sowie voraussichtliche Anzahl der aufzunehmenden Opfern [31]. Vorab definierte Ansprechpartner bei Polizei sowie bei den nichtpolizeilichen Organisationen dienen hierzu.

„Im Sinne einer effizienten Einsatzführung, in Anbetracht der möglichen Unübersichtlichkeit und Komplexität der Lage, aber vor allem aus Gründen einer gesellschaftlichen Gesamtverantwortung, kommt hierbei einer einheitlichen Koordination und Führung der in der nichtpolizeilichen Gefahrenabwehr beteiligten Behörden und Organisationen eine zentrale Bedeutung zu“ [7]. Empfehlenswert ist es in diesem Kontext, die etablierten Stabsmodelle, wie in den Dienstvorschriften für Polizei und Feuerwehr beschrieben [32], anzuwenden. Durch die Anwendung etablierter Strukturen ist, unabhängig von Größe und zeitlichem Verlauf einer Katastrophe, eine einheitliche Vorgehensweise aller beteiligten Behörden und Organisationen gewährleistet; Missverständnisse in der Kommunikation werden so weitgehend vermieden. Übergeordnet „erscheint für das Zusammenwirken aller beteiligten Behörden, Stellen und Organisationen der nichtpolizeilichen Gefahrenabwehr eine einheitliche Leitung unabdingbar; insbesondere vor dem Hintergrund, sowohl die Anzahl und Komplexität in den Melde- und Befehlsstrukturen als auch die damit verbundenen Kommunikationsbedürfnisse aller Beteiligten auf ein Mindestmaß reduzieren zu können“ [7]. Insbesondere in den Kliniken ist dieses Stabsmodell jedoch noch weitgehend unbekannt [33] und bedarf deshalb der Festschreibung im örtlichen Krankenhausalarm und -einsatzplan (KAEP) und auch dessen Beübung.

3. *Einsatzabschnitt Klinikum*
  *Ein geplanter Patiententransport erfolgt nur in Kliniken, die im sicheren Bereich liegen.*
  *Lageabhängig ist zur Aufrechterhaltung der Sicherheit in potenziell gefährdeten Kliniken, je nach Kräfteverfügbarkeit, die polizeiliche Durchsuchung aller eintreffenden Patienten, Betroffenen (unverletzt Beteiligter), Besucher und Mitarbeiter anzustreben.*
  *Als Mindeststandard ist auch bei eingeschränkter medizinischer Kräfteverfügbarkeit die Vorsichtung (auch durch nicht-ärztliches Personal) nach mSTaRT (Modified Simple Triage and Rapid Treatment) erforderlich. Sobald möglich soll eine qualifizierte ärztliche Sichtung aller Patienten erfolgen.*
  *Eine zusätzliche Unterstützung zur Aufrechterhaltung der Funktionsfähigkeit der Kliniken durch die jeweils zuständige Gemeinde kann sinnvoll sein.*

Bei einem Terroranschlag oder auch bei einem Amoklauf ist mit einer Vielzahl an Verletzten zu rechnen. Zudem ist ein „Second Hit“ sowohl am Anschlagsort oder auch in einer terroropferaufnehmenden Klinik zu befürchten [15]. Daraus folgt, dass sich die taktische Vorgehensweise am Ort des Geschehens ändern muss [34]. Bei einem konventionellen Großschadensereignis ist die Stabilisierung der Patienten vor Ort anzustreben. Hierzu errichten Rettungs- und Katastrophendienst neben einer Sichtungsstelle Behandlungsplätze ein, aus denen die Verletzten nach der Erstversorgung in festgelegter Reichenfolge abtransportiert und großflächig in Kliniken verteilt werden. Im Gegensatz dazu muss der Aufbau eines Behandlungsplatzes bei einem Terroranschlag wegen akuter Gefährdung von Patienten und Rettern am Anschlagsort hintanstehen [34]. Zu berücksichtigen ist auch das geänderte Verletzungsmuster durch penetrierende und perforierende Gewalteinwirkung, was eine suffiziente Erstversorgung am Schadensort erschwert oder ausschließt und den schnellstmöglichen Transport in eine Klinik gebietet [4]. Nach Wurmb [34] sind demnach taktische Einheiten, wie z. B. Behandlungsplätze am Schadensort, nicht angebracht, sondern das Ziel ist „clear up the scene immediately“. Hierbei ist zu beachten, dass Verletzte aus dem unmittelbaren Gefahrenbereich ausschließlich durch Einsatzkräfte der Polizei notversorgt und zu einem teilsicheren Bereich verbracht und dort dem Rettungsdienst übergeben werden [7]. Dies hat jedoch zur Folge, dass viele ungesichtete und unversorgte Patienten in schneller Reihenfolge in den nahegelegenen Kliniken eintreffen [15]. Im Gegensatz zum Anschlagsort, der als „unsichere Zone“ definiert wird, ist somit meistens die nächstgelegene aufnehmende Klinik als erste „sichere Zone“ anzusehen.

Besondere Lagen, z. B. Geiselnahmen, Bedrohungslagen oder Zugriffsmaßnahmen auf bewaffnete oder gewaltbereite Personen, erfordern ein abgestimmtes Vorgehen von Polizei, Rettungsdienst und ggf. eingesetzten Kräften der Gefahrenabwehr [35]. Die Verpflichtung zur Gefahrenabwehr für die kritische Infrastruktur „Klinik“ liegt hoheitlich bei den Bundesländern [36] und dementsprechend bei einem terroristischen Anschlag bei den Organen der Polizei. Folgerichtig ist in den „Hinweisen für nichtpolizeiliche Gefahrenabwehr bei Terror- und Amoklagen“ des Landes Baden-Württemberg auch zu finden: „Bei einem Einsatz im weitestgehend geschützten Bereich ist darauf zu achten, dass erkennbar bewaffnete Personen vor einer technischen oder medizinischen Rettung durch Polizeikräfte durchsucht werden. Gleiches gilt für verletzte Täter oder Tatverdächtige, die zusätzlich polizeilich begleitet werden“ [7]. Sinnvoll wäre damit, in dieser Situation eine Lösung anzustreben, dass jeder Verletzte vor der Einlieferung in die Klinik durch eine Polizeistreife auf Waffen, Sprengstoff oder andere gefährliche Gegenstände untersucht wird und die Aufnahme in die Klinik erst nach Freigabe gestattet ist.

Mit der zunehmenden Terrorgefahr auch in Deutschland wird vom Bundesamt für Bevölkerungsschutz und Katastrophenhilfe empfohlen, Klinikeinfahrten und -gelände zu überprüfen und abzusichern [37]. Wie vulnerabel eine ungeschützte Zufahrt zum Klinikgelände sein kann, wurde kürzlich von Twieg et al. gezeigt, indem durch einen unkontrolliert fahrenden Kleinbus „nur durch Glück“ nicht viele Mitarbeitenden der Klinik zu Schaden gekommen sind [46].

Wann immer möglich, soll am Anschlagsort eine Vorsichtung vorgenommen werden. „Auf die Möglichkeit der Delegation der Vorsichtung der Patienten auf das Rettungsdienstpersonal sei besonders hingewiesen“ [7]. Letzteres erscheint auch nötig, da durch Alarmierung der Klinikärzte zur Versorgung der eintreffenden Patienten in der Klinik kaum Notärzte am Anschlagsort zur Verfügung stehen werden [10]. Jede Verzögerung des Patiententransports in aufnehmende Kliniken durch Warten auf Sichtungsärzte würde dem Postulat „clear up the scene immediately“ entgegenstehen. Ansonsten erfolgt die Transportpriorität, soweit möglich, durch die Führungskräfte im Rettungsdienst [7]. Die qualifizierte Sichtung der Patienten wird durch Einrichten einer Sichtungsstelle entweder vor oder am Eingang der aufnehmenden Klinik vorgenommen [4, 9, 10, 15, 16].

Vorsichtung am oder in der Nähe des Anschlagorts und definitive Sichtung vor oder im Klinikbereich unterscheiden sich jedoch. Aus der Dringlichkeit „clear up the scene immediately“ und dem Gesichtspunkt der Transportpriorität kann zur Vorsichtung nur ein sehr vereinfachter Algorithmus, wie z. B. der mSTaRT, zur Anwendung kommen. Im Klinikbereich hingegen empfiehlt es sich, einen in der Literatur etablierten behandlungsorientierten Algorithmus wie den „Berliner Sichtungsalgorithmus“ anzuwenden [45].

4. *Kommunikation*
  *Es wird empfohlen, den Standard-Meldeweg (Führungs- und Lagezentrum der Polizei [FLZ]* *→* *Integrierte Leitstelle [ILS]* *→* *Klinik) auch im Falle einer LebEL zu verwenden, da die Verwendung alternativer, nicht regulär verwendeter Meldewege das Risiko von Übermittlungsfehlern hat. Darüber hinaus soll der Meldeweg für Sonderlagen (hier: LebEL) von der ILS zu den Kliniken definiert, beübt und 24/7 verfügbar sein.*
  *Das einheitliche Alarmstichwort „LebEL“ für alle ILS und Kliniken ist landesweit anzuwenden.*
  *Zusätzlich zum normalen Kommunikationsweg zwischen ILS und Klinik bei MANV wird ein Kommunikationsweg zur Polizei benötigt. Diese Kommunikation soll in erster Linie direkt persönlich vor Ort zwischen dem Verantwortlichen der Polizei vor Ort und dem medizinisch Verantwortlichen der Klinik sowie des Rettungsdienstes (in der Regel LNA) erfolgen. Eine gegenseitige telefonische Erreichbarkeit soll gegeben sein. Rückfallebenen im Falle eines Ausfalls von Mobilfunknetzen sollten eingeplant werden, zum Beispiel durch die Definition eines Treffpunkts und einer Uhrzeit im Vorfeld.*
  *Für den Gesamteinsatz sollten zur optimalen Zusammenarbeit zwei Kommunikationsebenen etabliert werden:*
    *Stabsebene zwischen Polizeiführer und LNA (mit Stabsqualifikation)*
    *Führungsebene (gemeinsame Befehlsstelle) unmittelbar vor Ort (bei mehreren Örtlichkeiten auch mehrfach)*

Als übergeordnetes Ziel bei der Planung der Maßnahmen zur Versorgung von Patienten bei LebEL ist die „reibungslose und koordinierte Zusammenarbeit aller beteiligten Stellen auf Basis einer transparenten und durchgängigen Kommunikation“ zu nennen [7]. Dabei ist das Zusammenwirken aller beteiligten Organisationen unabdingbar. Damit die komplexe Kommunikation, die zudem noch oft analog und digital differiert und auch unterschiedlichen Melde- und Befehlsstrukturen folgt, den erhöhten Bedürfnissen eines Terroranschlags gerecht wird, sollten klar definierte Übermittlungswege im Vorfeld festgelegt werden. „Von besonderer Bedeutung ist die frühzeitige Kommunikation der Führungskräfte vor Ort“ [8]. Hierfür ist es u. a. notwendig, dass die Führungskräfte von Polizei, Feuerwehr, Rettungsdienst und Katastrophenschutz entsprechend gekennzeichnet sind. Während dies bei den nichtpolizeilichen BOS in aller Regel der Fall ist, z. B. „Leitender Notarzt“, ist die Führung der Polizei nicht ohne Weiteres zu erkennen. Hier sollte auch eine entsprechende Kennzeichnung eingeführt werden.

Ebenso wichtig ist aber auch die rückwärtige Kommunikation. Hier können die unterschiedlichen Kommandostellen von Polizei und nichtpolizeilichen Einsatzkräften problematisch werden. Einerseits laufen polizeiliche Meldewege über das Führungs- und Lagezentrum der Polizei, während Rettungsdienst und Feuerwehr durch die Integrierte Leitstelle repräsentiert werden. Zudem erhalten die nachgeschalteten aufnehmenden Kliniken meist zu wenig oder kaum Informationen über Geschehen, Umfang und Verletzungsgrad der aufzunehmenden Patienten [31]. Wie schon erwähnt, könnte eine gemeinsames Lagezentrum, in dem Polizei und BOS vertreten sind, die Kommunikation und den aktuellen Informationsstand verbessern und zudem die Kliniken mit regelmäßigen Informationen versorgen, damit diese dem jeweiligen Sachstand entsprechend organisatorische und operative Kapazitäten anpassen könnten.

- *Für die Vorplanungen ist die Einrichtung eines hauptamtlichen bestellten Verantwortlichen für Klinikalarm- und Einsatzplanung für Sonderlagen in den größeren Kliniken (überregionales Traumazentrum) zu empfehlen. Für alle anderen Kliniken sollte ein entsprechender Verantwortlicher mindestens im Nebenamt bestellt werden.*

Vorplanung setzt das Erkennen von Risiken und deren Management voraus. Für eine Risikoanalyse sind ausreichend Ressourcen (Material, Personal und Zeit) einzuplanen [37]. Das reine Reflektieren der potenziell in Betracht kommenden Maßnahmen zum Schutz vulnerabler Infrastrukturen ist angesichts der Komplexität der Finanzierung des deutschen Gesundheitssystems und seiner in Teilen anarchisch organisierten Krankenhauslandschaft nicht ausreichend. Die anstehenden Planungs- und Koordinierungsaufgaben sind äußerst umfangreich. Die Entwicklung einer leistungsstarken Infrastruktur zur Bewältigung sich ständig ändernder Gefahrenlagen bedarf der Einbindung der gesamten verfügbaren Expertise [38]. Zudem sind die Kliniken per Gesetz verpflichtet, zur Mitwirkung im Katastrophenschutz für ihre Krankenhäuser Katastropheneinsatzpläne aufzustellen und fortzuschreiben; diese sind mit den Katastrophenschutzplänen der Katastrophenschutzbehörden abzustimmen [39]. Auch in großen Kliniken wird oft Katastrophenschutz bzw. der Katastrophenschutzbeauftragte als nebenamtlich ausreichend verstanden. Risikoanalyse, Vorplanung, Alarmplan-Aktualisierung sowie die Notwendigkeit zu Übungen können jedoch in nebenamtlicher Tätigkeit nicht ausreichend wahrgenommen werden. Für eine suffiziente Gefahrenabwehr ist deshalb zumindest an großen Kliniken ein hauptamtlich Verantwortlicher zu bestellen. Sinnvollerweise können diese Aufgaben mit dem Amt des Katastrophenschutzbeauftragten der Klinik verbunden werden.

5. *Sicherheit der Infrastruktur Klinikum*
  *Derzeit bestehen in den meisten Kliniken weder die baulichen noch die personellen Voraussetzungen zur Gewährleistung des Schutzes eines Klinikums gegenüber gewaltbereiten Tätern.*
  *Es wird empfohlen, für jede Klinik ein an die lokalen Gegebenheiten angepasstes Sicherheitskonzept (baulich, technisch, organisatorisch) zu erstellen.*
  *Aufgabe der lokalen Sicherheitskonferenz ist es, Schwachstellen zu analysieren und für die LebEL die Erstellung entsprechender Konzepte (gem. der Aufgabenzuweisung zwischen polizeilicher und nichtpolizeilicher Gefahrenabwehr) zu veranlassen.*
  *Die Wahrnehmung von Sicherheitsaufgaben in einer Klinik ist Kernelement der Gewährleistung der Verletztenversorgung (siehe auch „Schutz kritischer Infrastrukturen (KRITIS)“). Die Etablierung eines funktionsfähigen, von der jeweiligen Klinik gestellten Sicherheitsdienstes, der bei Bedarf kurzfristig aufwachsen kann (Alarmierung), ist wünschenswert.*
  *Lageabhängig übernimmt die Polizei eine Funktionssicherung der kritischen Infrastruktur Klinikum (Freihalten von Rettungswegen, Sicherstellung der Patientenversorgung, Lenkung von Angehörigen und Presse). Schwerpunkte sind bei einer LebEL der Sichtungsbereich und der Betreuungsbereich. Je nach Lage und Kräfteverfügbarkeit wird die Polizei Sicherheitsaufgaben an Kliniken nicht oder nur begrenzt wahrnehmen können. Die Zusammenarbeit zwischen der Polizei und dem Sicherheitsdienst der Klinik soll abgestimmt und kooperativ erfolgen.*

Ein potenziell gravierender Unterschied besteht zwischen „normalem“ MANV und terroristischen Anschlägen bezüglich der Sicherheitslage in Kliniken. Krankenhäuser können, wie schon dargestellt, durch einen „second hit“ gefährdet sein. Da bei einem Terroranschlag die meisten Patienten schwer verletzt und ungesichtet in die Klinik kommen, können darunter auch bewaffnete Attentäter sein, oder aber das Krankenhaus ist per se ein zweites Angriffsziel, um die Versorgung der Terroropfer unmöglich zu machen. Das „National Counter Terrorism Security Office“ teilte 2017 in einem Dokument mit, dass mehrere Terrororganisationen bereits Krankenhäuser als potenzielle Angriffsziele identifiziert hätten [40]. Krankenhäuser müssen daher entsprechende Vorkehrungen treffen [36].

Krankenhäuser zählen per definitionem zur kritischen Infrastruktur eines Landes, deren Ausfall oder Beschädigung zur Gesundheitsgefährdung der Bevölkerung führen kann. Zwar halten einige größere Kliniken zum Schutz des Personals, v. a. in den Notaufnahmen, in begrenztem Maße Sicherheitspersonal vor. Dieses ist in keiner Weise jedoch in der Lage, eine Klinik wirkungsvoll gegen terroristische Attacken zu schützen. Es ist deshalb zu fordern, dass Krankenhäuser ein innerklinisches Sicherheitskonzept erarbeiten, pflegen und fortentwickeln. Teilnehmer*innen der 3. Notfallkonferenz der Deutschen Gesellschaft für Unfallchirurgie wurden nach dem Vorhandensein eines Sicherheitskonzepts in ihrer Klinik befragt. 34 % bejahten das Vorhandensein eines Sicherheitskonzepts, während 38 % dies verneinten. 28 % der Befragten war dies unbekannt. Auf Übungen mit terroristischen Inhalten angesprochen, gaben nur 26 % an, dies zu berücksichtigen [36]. Zudem gibt die Publikation keine Hinweise über Ausmaß und Wirksamkeit des Sicherheitskonzepts. Dies deckt sich mit einer Umfrage an 214 Kliniken in Baden-Württemberg, bei der nur 26 % angaben, dass sie im KAEP terroristische Gesichtspunkte berücksichtigen würden [31]. Diese Zahlen lassen vermuten, dass wahrscheinlich weniger als ein Drittel der deutschen Krankenhäuser zumindest in einem gewissen Maße Abwehrmaßnahmen gegen eine „second hit“ ergreifen könnten.

6. *Ausbildung und Übungen*
  *Es wird die Erarbeitung eines abgestimmten Aus- und Fortbildungskonzepts als modulares System für Polizei, Kliniken und nichtpolizeiliche Gefahrenabwehr empfohlen.*
  *Schulungen sollten regelmäßig und zielgruppenangepasst stattfinden, ggf. mit organisationsübergreifenden Referenten.*
  *Übungen sollten regelmäßig stattfinden. Eine besondere Bedeutung kommt organisationsübergreifenden Übungen zu, hier liegt eine der Aufgaben der lokalen Sicherheitskonferenz.*

Modulare Aus- und Fortbildungskonzepte haben sich in vielfältigen medizinischen und nichtmedizinischen Bereichen bewährt [41]. Schulungen und Übungen vermitteln nicht nur aktuelles Wissen, sondern der Lernerfolg wird auch praktisch überprüft. So können Katastrophenübungen dazu beitragen, dass Lücken in Notfallplänen und -verfahren identifizieren werden, die, wenn sie behoben werden, zur Verbesserung der Notfallvorsorge des Systems führen [42].

Die „Entwicklung von Standards und Leitlinien für die Aus- und Weiterbildung in der multidisziplinären Gesundheitsreaktion auf Großereignisse, die den Gesundheitszustand einer Gemeinschaft bedrohen“ hat in den USA in den letzten Jahren an Bedeutung gewonnen [43]. So wird die Notwendigkeit einer schnellen und effektiven Ausbildung des Gesundheitspersonals auf allen Ebenen mittlerweile von der Joint Commission on Accreditation of Healthcare Organizations (JCAHO) allgemein anerkannt und empfohlen [43]. Für eine wirksame Katastrophenhilfe ist zu fordern, dass medizinisches Personal nicht nur über Wissen, sondern auch über spezifische technische Fähigkeiten und Entscheidungsfähigkeiten verfügen sollte. Ein kompetenzbasierter Ansatz könnte den Rahmen für die Durchführung dieser Art eines flexiblen Trainings bilden [44]. Das vom Bundesamt für Bevölkerungsschutz und Katastrophenhilfe empfohlene Handbuch zur „Krankenhausalarm- und -einsatzplanung“ [37] skizziert wenigstens einen Orientierungsrahmen zur Erstellung eines einheitlichen KAEP, eine bundesweite Verpflichtung zu einer einheitlichen Gestaltung der Planung oder gar Inhalte zu Schulung und Übungen fehlen jedoch nach wie vor. Gravierender wirkt sich aus, dass Standards für präklinische Katastrophenübungen oder gar zur Schnittstelle zwischen Präklinik und Klinik damit nicht existieren.

Forderungen nach Schulung und Übungen für einen Terroranschlag stehen und fallen mit der Frage nach der Finanzierung. Erst die Klärung der Finanzierungsfrage ebnet den Weg für ernsthafte Bedarfsplanungen und die Entwicklung konkret fassbarer Konzepte. Übungen sind kostenintensiv, für die sowohl Bund, Länder wie auch Versicherungsträger keine Finanzierung zur Verfügung stellen. Damit bleibt nur, dass die Klinikbetreiber die aus ihrem Versorgungsauftrag ableitbaren Opportunitätskosten für die Vorsorge auf einen Terroranschlag selbst tragen. Da hierbei nahezu unvermeidbare Qualitätsdefizite auftreten, unterstreicht dies die Dringlichkeit einer zeitnahen Klärung der Finanzierungsfrage [38].

## Fazit für die Praxis

- Zu fordern sind kompetenzbasierte Schnittstellenlösungen für ein reibungsloses Zusammenwirken von Präklinik und Klinik bei der Versorgung von Patienten, die Opfer einer LebEL wurden.
- Für die Vorplanung wird die Einrichtung einer lokalen Sicherheitskonferenz auf der Ebene Landkreis/Untere Katastrophenschutzbehörde mit folgenden Teilnehmern empfohlen: Untere Katastrophenschutzbehörde, Feuerwehr, regionales Polizeipräsidium, Leitender Notarzt, Rettungsdienste, Katastrophenschutzbeauftragte betroffener Kliniken.
- Empfohlen wird, ein gemeinsames Führungs- und Lagezentrum (FLZ) einzurichten, wo sich Führungspersonal von Polizei, Rettungsdienst, Feuerwehr und Katastrophenschutz einfinden, um die Abwicklung des Schadensereignisses gemeinsam, kompetent und ohne Zeitverlust zu organisieren. Aus diesem FLZ sollte dann ein „Verbindungsbeamter“ die Kliniken in regelmäßigen zeitlichen Abständen mit Informationen versorgen.
- Übungen sollten regelmäßig stattfinden. Eine besondere Bedeutung kommt organisationsübergreifenden Übungen zu; hier liegt eine der Aufgaben der lokalen Sicherheitskonferenz.
